# Application of Janus Particles in Point-of-Care Testing

**DOI:** 10.3390/bios12090689

**Published:** 2022-08-26

**Authors:** Yuhan Wang, Peixuan Zhao, Shihao Zhang, Kexiao Zhu, Xiaoya Shangguan, Lishang Liu, Shusheng Zhang

**Affiliations:** Shandong Provincial Key Laboratory of Detection Technology for Tumor Markers, Linyi University, Linyi 276005, China

**Keywords:** janus particles, point-of-care testing, biosensors, detection

## Abstract

Janus particles (JPs), named after the two-faced Roman god, are asymmetric particles with different chemical properties or polarities. JPs have been widely used in the biomedical field in recent years, including as drug carriers for targeted controlled drug release and as biosensors for biological imaging and biomarker detection, which is crucial in the early detection and treatment of diseases. In this review, we highlight the most recent advancements made with regard to Janus particles in point-of-care testing (POCT). Firstly, we introduce several commonly used methods for preparing Janus particles. Secondly, we present biomarker detection using JPs based on various detection methods to achieve the goal of POCT. Finally, we discuss the challenges and opportunities for developing Janus particles in POCT. This review will facilitate the development of POCT biosensing devices based on the unique properties of Janus particles.

## 1. Introduction

Micro/nanoparticles with anisotropic physicochemical properties are called Janus particles (JPs) [1,2,3]. The term comes from the name of the ancient Roman god Janus, who had two different faces. Veyssie and his colleagues created glass beads with hydrophobic and hydrophilic sides in 1989, which they then used to create the first Janus nanoparticles [4]. Subsequently, many methods to prepare JPs have been proposed [5]. Janus particles, as new particles, have attracted extensive research interest [6,7]. JPs with dual functions produced by chemical heterogeneity, due to their potential applications in electronic paper [8], photonic materials [9], emulsion stabilization [10], imaging probes [11], and sensors, have attracted extensive attention [12,13]. Due to the different physical and chemical properties of JPs, different reactions can occur on both sides of asymmetric JPs, producing unequal propulsion force so that the nanoparticles have a particular direction of propulsion force [14,15,16,17]. JPs provide a new idea for the movement of particles and have been widely used as drug carriers [18,19,20] and to build biosensors [21,22].

Diagnostic tools used at the point of care (POC) are crucial to the health care system, especially for diagnosing and tracking illnesses [23,24,25]. Point-of-care testing (POCT) refers to testing “at the time and place of patient care”. Unlike the traditional detection format, POCT should be friendly to users and patients [26]. POCT has several benefits, such as the capacity to deliver quick and precise results, convenience of use, low cost, and little requirement for specialist equipment [27,28,29]. POCT devices can be divided into small handheld devices and large benchmark devices. Small handheld devices are combined with micro/nanoparticles, including paper-based microfluidics and printed electrode (PE) POCT devices [30,31]. These devices provide a wide range of qualitative or quantitative measurements for analysis [32,33]. Creating a chip-based [34], miniature [35], transportable [36], and self-contained device [37] that analyzes various analytes in intricate samples is one of the objectives of POCT [38,39,40]. POCT represents a paradigm change from traditional diagnostic tests in the clinical laboratory environment to near-patient settings, enabling doctors to make more informed decisions about diagnosis and treatment by giving them access to rapid diagnostic information.

**Figure 1:**
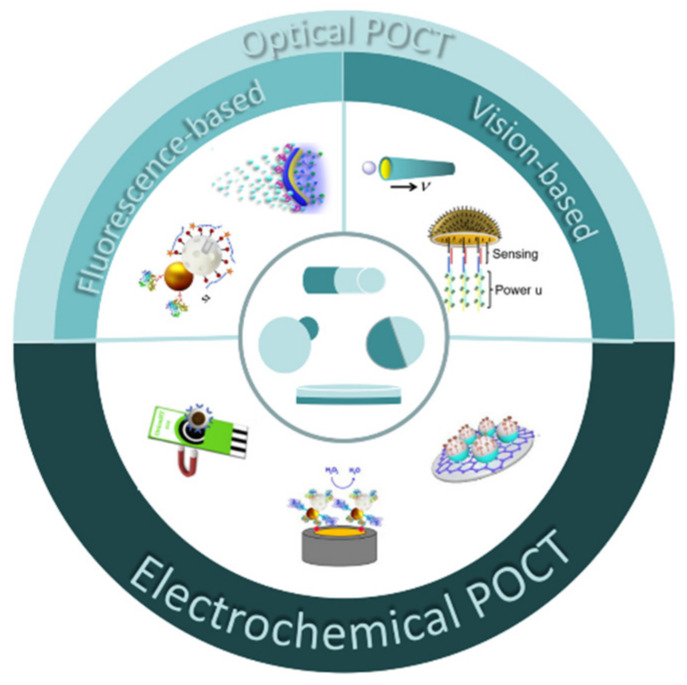
Schematic illustration of typical Janus particles with different surface properties and various applications.

JPs have been widely used in drug delivery [41,42,43] and in vivo imaging [44,45], especially in tumor therapy [46,47]. The exceptional qualities of nanoparticles can be fully utilized when JPs are added to POCT [48,49,50]. The asymmetric modification of particles can provide great convenience and a more comprehensive application prospect for detection [51,52,53,54]. For the precise and sensitive detection of aspartic acid in mouse brains, colorimetric analysis of gold nanoparticle aggregation based on ethylene glycol (PEG) and highly selective recognition agent cysteine (CYS) has been used in the past [55]. In this review, we first discuss the preparation methods of JPs, followed by the application in biological detection, including electrochemical, fluorescence, and visual detection (Figure 1). We firmly believe that JPs can be widely used in POCT soon.

## 2. Preparation of Janus Particles

Janus particles can be produced in various morphologies and geometries [56,57,58]. Asymmetric modification on micro/nanoparticles is a significant problem in synthesizing JPs [59,60,61], and many different preparation methods have been developed for this problem [62,63,64]. Their synthesis methods can be divided into the following four categories [65,66]: the microfluidic method, sputtering method, phase-separation method, and Pickering emulsion method [67,68,69]. We will go over a few of these techniques as a general introduction, because discovering new uses for Janus particles requires understanding how they are created.

### 2.1. Microfluidic Method

Microfluidics is the science and technology used to process or manipulate tiny fluids with volumes between nanoliters and microliters (sizes from tens to hundreds of microns) [70,71]. It is a brand-new multidisciplinary field that combines biology, biomedical engineering, microelectronics, fluid physics, chemistry, and novel materials [72]. Microfluidic devices [73], also known as chip laboratories and microtonal analysis systems [74], are typically referred to as microfluidic chips due to their downsizing and integration [75,76]. 

**Figure 2:**
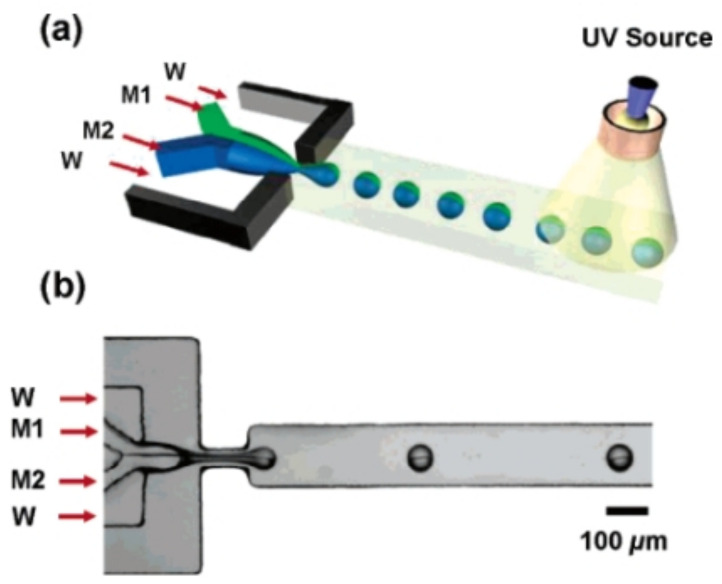
(**a**) Schematic of the generation of Janus droplets from immiscible monomers M1 and M2, emulsified in an aqueous solution of SDS (W). The droplets are irradiated with UV light in the downstream channel. (**b**) Optical microscopy image of the formation of Janus droplets. M1, M2, and W flow rates are 0.02, 0.02, and 4 mL/h, respectively. Reprinted with permission from ref. [62]. Copyright © 2006 American Chemical Society.

Microfluidics can prepare large Janus particles by pumping two different polymer fluids into various inlets of the device in a charged “Y” microfluidic device [62]. The two liquids aggregate and adhere during flow in the widget to form Janus particles (Figure 2). There are two standard methods to prepare Janus particles based on microfluidics [77]: one is to prepare particles using droplets as templates, and the other is to prepare particles by direct curing through masks [78]. For the former method, Janus droplets are generally formed using microfluidics first, and then the droplets are cured into Janus particles. Janus droplets can be formed on a chip by constructing a flow-focused structure. The synthesis of particles with complex morphology, such as multiphase asymmetric particles, using this method requires three-dimensional geometry of the channel and precise control of the multiphase liquid flow [79]. For the latter type of method, lithography and microfluidic multiphase laminar flow techniques are often combined to form two or more phases of laminar flow within the microchannel of the chip, cover the channel with a mask of a set shape, and solidify the particles within the medium by means of lithography, with the formation of the particles depending on the conditions shown.

**Figure 3:**
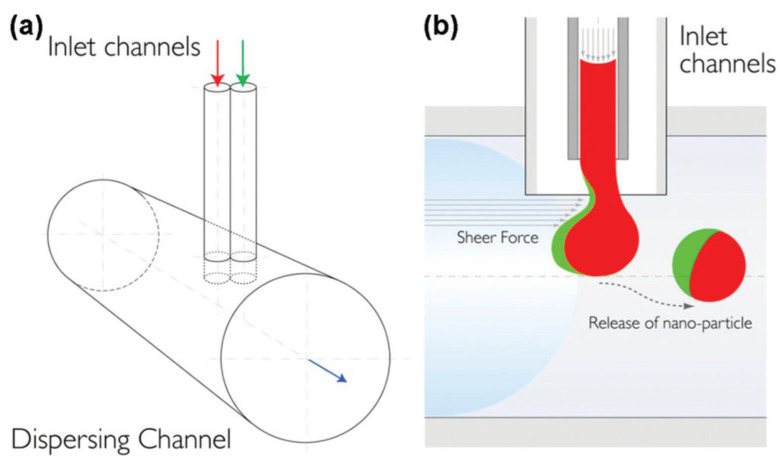
Schematic of the modified fluidic nanoprecipitation system (FNPS). (**a**) Cartoon of FNPS. Sample inlets are inserted into the dispersing channel via a “T” connector. The inlet channels contain two PLGA polymers that make contact at the exit of the inlet streams and precipitate upon contact with the surfactant in the dispersing channel, solidifying the particles. (**b**) Side view of the channels. PLGA droplets are exposed to the hydrodynamic force of the continuous flow. Reprinted with permission from ref. [80]. Copyright © 2012 American Chemical Society.

Xie et al. were the first to report the use of Janus polymeric NPs to combine a hydrophobic medicine (paclitaxel-PTX) and a hydrophilic drug (adriamycin hydrochlorid—dox) in a single particle [80]. Two compartments of a fluidic nanoprecipitation system containing various forms of poly(lactic acid-protoethanolic acid) (PLGA) were used to create Janus polymeric NPs (Figure 3). These two medications were chosen because their systemic delivery was troublesome and could be improved by encapsulating them. The microfluidic synthesis method has high mass and heat transfer efficiency, easy and accurate control of reaction conditions, fast mixing speed, and low reagent consumption. The use of microfluidics for synthesizing Janus particles is beneficial in controlling the size and morphology of the particles [81], the synthesized particles have high homogeneity, and the preparation process is simple [82].

### 2.2. Sputtering Method

Sputtering is a simple method for preparing solid Janus nanoparticles [83]. The sputtering method is a modern technological method that uses sputtering principles and techniques to treat the surface of processed materials [84]. Sputtering works by bombarding the surface of a cathode target material with an ion stream created under the influence of a DC or RF high voltage electric field. This causes the atoms on the solid’s surface to fly out (or sputter) as a result of chemical bonds being broken [85].

**Figure 4:**
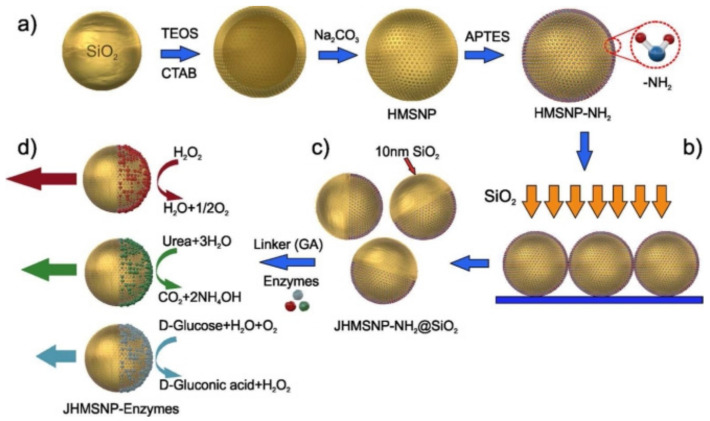
Fabrication of enzymatic hollow mesoporous silica Janus nanomotors. (**a**) Synthesis of HMSNP by using solid SiO_2_ nanoparticles as a template, and further surface modification of amino groups by the grafting method to produce HMSNP--NH_2_; (**b**) fabrication of JHMSNP-NH_2_@SiO_2_ by electron beam (e--beam) evaporation of SiO_2_ (10 nm) on a monolayer of HMSNP--NH_2_ and (**c**) detachment of the Janus nanoparticles by sonication treatment; (**d**) conjugation of enzymes onto one face of the Janus nanoparticles via a glutaraldehyde (GA) linker molecule. Reprinted with permission from ref. [87]. Copyright © 2015 American Chemical Society.

In the synthesis of Janus nanoparticles by sputtering, solid nanoparticles are uniformly distributed on a plane by evaporation to form a tight surface [86]. For example, a solution of monolayer silica particles was added dropwise to an oxygen plasma-treated slide, and a monolayer silica layer was formed on the decline by vacuum evaporation [87]. Subsequently, the metal is sputtered at a fixed angle (Figure 4). Due to the close alignment between the silica particles, the metal is deposited only on the exposed hemispheres of the silica particles. The prepared Janus silica particles can be dispersed off the slide by ultrasonication.

Using a tuning process, the metal layer’s thickness and the metal film’s shape can be altered when the sputtering technique is used to create JPs [88]. The sputtering method is easy and expedient for a wide range of nanoparticles, but only when a single layer of nanoparticles is formed on the substrate and the bottom of the nanoparticles is protected from the metal vapor by the substrate [89]. The sputtering method is suitable for synthesizing larger size particles, and the number of particles synthesized is relatively small and cannot be used for large-scale synthesis.

### 2.3. Phase-Separation Method

The phase-separation method is when a non-solvent or undesirable solvent, coagulant, or coagulation inducer is added to a mixed solution of core and wall materials [90], or the polymer’s solubility is reduced by changing the temperature [91] or pH [92], and it emerges out of the solution [93,94]. It is deposited on the surface of the wrapped core material to form microcapsules [95]. The nanoparticle solution and the non-aqueous emulsion droplets of the polymer blend evaporate in the solvent to create Janus particles [96,97]. The stable polymer of nanoparticles plays a crucial role in the anisotropic localization of nanoparticles in Janus particles and is only compatible with a polymer blend for phase separation [98]. Using this phase separation-based technique, excellent control of particle size, size distribution, and morphology may be attained [99]. 

**Figure 5:**
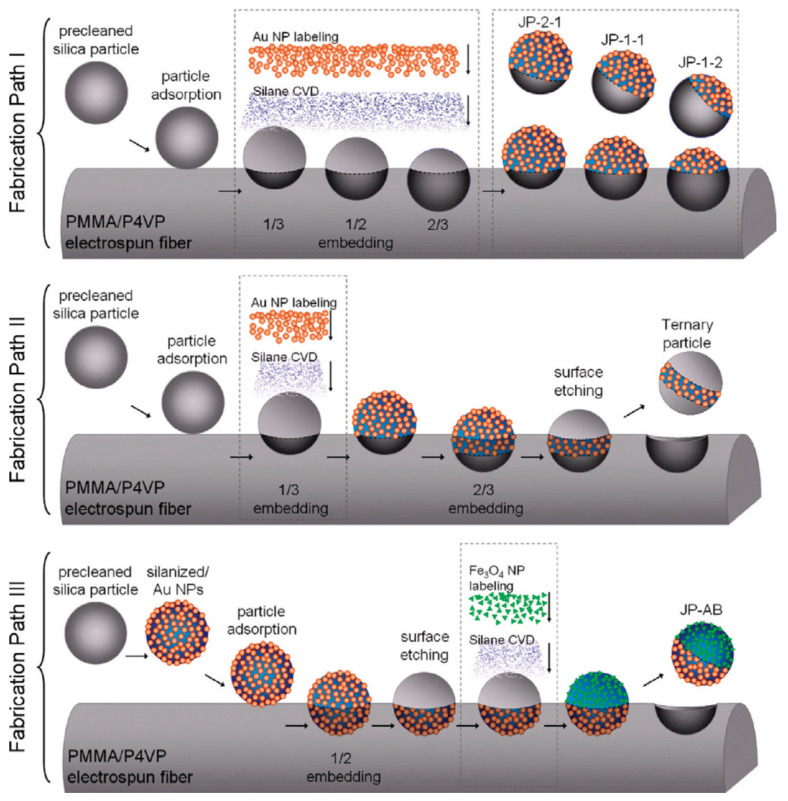
Three fabrication paths for (I) three kinds of asymmetric Janus particles, (II) ternary particles, and (III) biofunctionalized Janus particles. Reprinted with permission from ref. [100]. Copyright © 2010 American Chemical Society.

Using a sequentially aligned particle embedding and surface modification procedure, Kuo and colleagues created a series of asymmetric Janus silica particles [100]. Thermally induced particle embedding in polymeric fiber substrates allows for the fine control of the level of particle submergence and subsequent chemical alteration of the hemispherically exposed particle surface. Thermal manipulation controlled the embedding depth to embed the nanoparticles two-thirds, one-half, one-third, etc., of the way into the polymer (Figure 5). Then, Janus particles with different functional hemispheres were obtained at the desired ratio after labeling the amino-enriched surface with Au nanoparticles.

### 2.4. Pickering Emulsion Method

The Pickering emulsion is a dispersion system composed of two incompatible liquid phases in which the inner or dispersed phase is dispersed as droplets in the outer or continuous phase, i.e., [101], amphiphilic surfactants are not required to reduce interfacial tension [102], and stable emulsions can be produced by directly dispersing colloidal particles in the emulsion in the colloidal size range [103,104,105]. 

**Figure 6:**
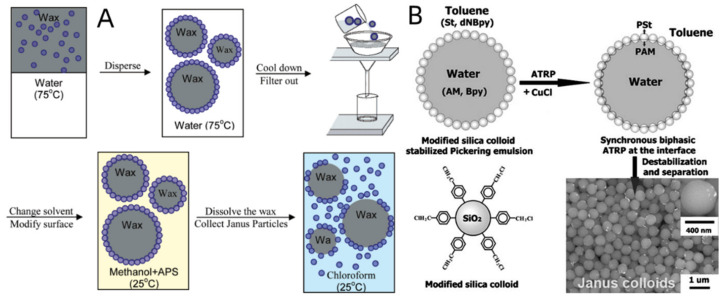
(**A**) Schematic procedure to create Janus particles by functionalizing particles adsorbed onto an emulsion of water and oil and then cooling the sample so that the oil crystallizes to form a wax. Reprinted with permission from ref. [108]. Copyright © 2006 American Chemical Society. (**B**) Synthesis of Janus colloids by biphasic grafting at a Pickering emulsion interface. Reprinted with permission from ref. [109]. Copyright © 2008 WILEY--VCH.

When making JPs using the Pickering emulsion technique, solid particles can stabilize the liquid–liquid emulsion to create a Pickering emulsion in which the particles are immobilized at the liquid–liquid interface to reduce the overall surface energy of the solution [106,107]. Granick mixed silica particles with paraffin wax to create a solution, and then added water to develop JPs. He was one of the first pioneers in applying this approach [108]. This encouraged the silica NPs to be trapped and locked in the oil–water interface, where they were then subjected to chemical modification to produce JPs that were partially cationic or anionic (Figure 6A). The Pickering emulsion method is favorable for preparing Janus nanoparticles by selective conversion because it can usually create a doubly different chemical environment for the solid particles. For instance, Liu et al. observed that silica colloidal surfaces simultaneously underwent the transition of two additional characteristics [109]. The oil–water interface was where the silica colloid was found. The hydrophobic and hydrophilic sides of the silica colloid each have a distinct local environment during the process (Figure 6B). Hydrophobic polymer brushes can be altered on the silica colloid’s exterior during the polymerization process, but hydrophilic polymers can only be affixed to the interior [110,111]. The Pickering emulsion technique is frequently used to create controlled-morphology Janus nanoparticles of different sizes and compositions [112]. The size and wettability of the nanoparticles have a big impact on how much of the surface area is on the oil side vs. the water side [113]. The two characteristics of Janus particles produced by the Pickering emulsion method have different hydrophilic and lipophilic properties [114], providing significantly improved adhesion to materials and solving the weak adhesion problems of traditional hydrophobic coatings [115].

This study explores several major synthesis methods of JPs. Design strategies for the morphology, particle size, composition, and surface modification of JPs are explored, which in turn affect the performance of JPs [116]. Table 1 summarizes the typical fabrication methods, compositions, particle size and morphologies of several representative Janus structures. For (sub)micron-sized JPs, structural asymmetry can be directly visualized using conventional electron microscopy. However, for smaller, nanoscale Janus particles, explicit characterization and validation become more challenging [117]. TEM is commonly used in the laboratory to characterize Janus nanoparticles with sufficient contrast differences in both compartments, and elemental analysis can also be used to represent different element species on both sides of the JPs [118]. When JPs are composed of two polymers, it is necessary to further stain the polymers selectively to characterize the morphology of JPs [119]. Therefore, future progress requires an adequate description of JPs to characterize the anisotropy of JPs.

## 3. Janus Particles for Electrochemical POCT

Electrochemical biosensors have become essential resources for building portable [127,128,129,130,131] and patient-focused care devices [132,133,134,135] due to their comparatively low detection limit [136], straightforward use, and real-time detection of biomarkers [137,138,139]. Most electrochemical biosensors used in cancer detection and monitoring are aptamer-based [140,141,142,143]. The inherent benefits of aptamer-based electrochemical biosensors include excellent stability [144], specificity [145], comparatively low cost [146], and the ability to build the hairpin-like structures necessary for these biomolecules’ analysis [147,148]. A complicated arrangement on the electrode surface can be created using aptamers and sophisticated JPs [149] for easy and accurate cancer biomarker quantification [150,151,152,153]. The anisotropy of JPs is used to modify them asymmetrically. One side is modified with aptamers or biological enzymes to capture biomarkers, and the other side is amplified or combined with electrodes to complete electrical signal conduction.

**Figure 7:**
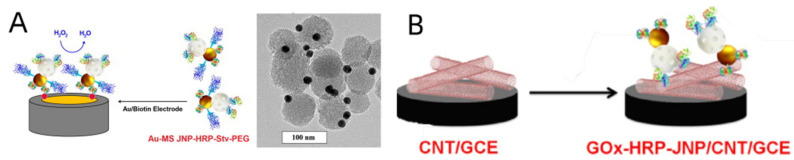
(**A**) Au MS JNPs dually functionalized with HRP and Stv and polyethylenglycol chains used as a biorecognition signaling system or GOx and HRP enzymes as electrode modifiers for affinity and catalytic electrochemical biosensing, respectively. Reprinted with permission from ref. [125]. Copyright © 2013 Elsevier B.V. (**B**) Schematic display of the steps involved in the assembly of the GOx–HRP--JNP/CNT/GCE bienzyme biosensor. Reprinted with permission from ref. [154]. Copyright © 2015 WILEY--VCH.

The utilization of Au-mesoporous silica Janus nanoparticles (AuMS JNPs) and biofunctionalized JPs for catalytic and accurate electrochemical biosensing was reported by Villalonga’s group [125]. The first study discussed AuMS JNP, which covalently immobilized horseradish peroxidase (HRP) and was doubly functionalized with streptavidin (Stv) and polyethylene glycol chains. HRP was used as the enzymatic signal transduction component on a mesoporous silica surface, and Stv and PEG chains were used as biometrics and solubilizers on a Au surface to achieve the purpose of immobilizing JPs to Au electrodes. (Figure 7A). By detecting HRP reduction of H_2_O_2_ using cyclic voltammetry (CV), this functionalized JP with biometric signal transduction ability was successfully used to identify biotin on a gold surface. The same team reported engaging carbon nanotubes (CNTs) as GCE modifiers along with AuMS JNPs and glucose oxidase (GOx) two years later [154]. HRP was mounted on the Au and mesoporous silica faces to create a glucose biosensor (Figure 7B). The biosensor was used to analyze the amount of glucose in commercial soft drinks and had a LOD of 360 nM.

**Figure 8:**
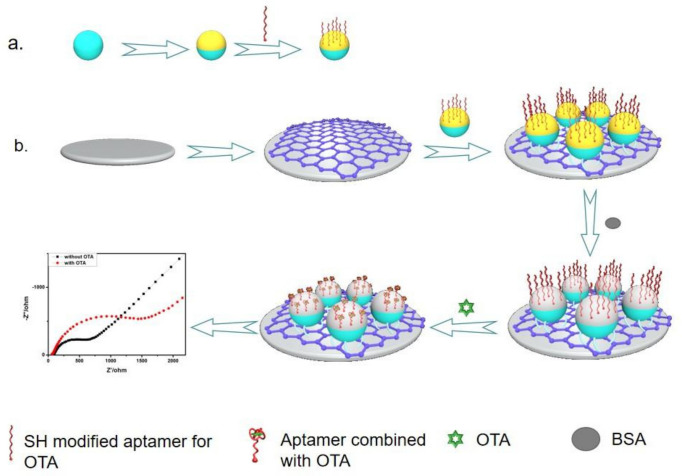
Synthesis strategy of Janus particles (**a**) and the schematic diagram of the sensing principle of the ochratoxin A immunosensor (**b**). Reprinted with permission from ref. [155]. Copyright © 2019 Elsevier B.V.

In a relatively new technique, aptamer/NH_2_ JPs can be used for the electrochemical determination of ochratoxin A (OTA) [155]. Ochre mycotoxin A is a significant threat to human health and is extensively prevalent in various foods, including coffee beans, spices, and cereal items. Ochratoxin A is highly hepatotoxic and nephrotoxic and has teratogenic, mutagenic, and carcinogenic effects, according to toxicological tests. The surface of the amino styrene particles is coated with gold, followed by fixation of the aptamer combined with OTA on the gold surface, and the other hemispherical is able to bind to the glassy carbon electrode via a peptide bond (Figure 8). The interaction between aptamers and OTA can be captured by differential pulse voltammetry. Similar LOD values were obtained by two aptamers (3.3 × 10^−14^ and 1.0 × 10^−14^ M, respectively) in GCE chemically modified with carboxylated graphene (COOH-GN) by carbon diamide/succinimide (EDC/NHS), which are competitive with previously published methods. Aptamers are used to examine human urine samples (RAC) and wine (OTA) samples.

**Figure 9:**
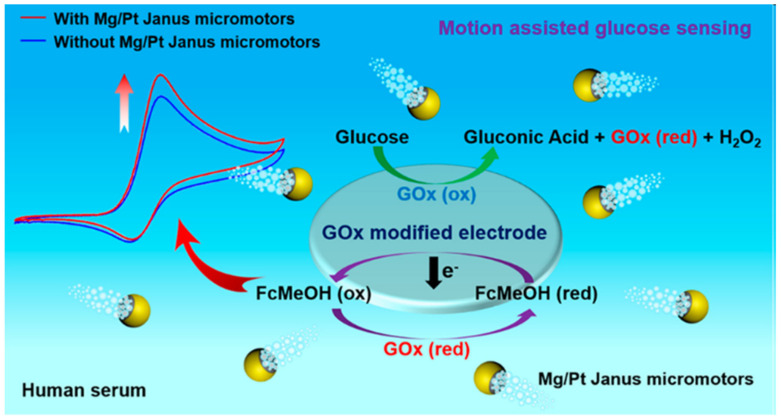
Schematic representation of Mg/Pt Janus micromotor-assisted glucose biosensing in human serum using screen--printed electrodes. Reprinted with permission from ref. [156]. Copyright © 2019 American Chemical Society.

Certain JPs’ efficient self-propulsion enables them to mix with liquids considerably better than static particles, improving the efficiency of dynamic electrochemical detection without external stirrers. For instance, Kong et al. combined the Mg/Pt Janus micromotor with cyclic voltammetry to detect glucose in human serum [156]. The system follows a second-generation glucose biosensor principle where a mediator, FcMeOH, was introduced to facilitate the heterogeneous electron transfer more efficiently upon the enzymatic breakdown of glucose by GOx. At the optimal Mg/Pt Janus microkinetic concentration, a linear relationship between the current signal and the glucose concentration was established. The LOD was 3.32 × 10^−2^ M in the presence of a 1 mg/mL micromotor. Through the buffering of chloride and plasma in human serum, pit corrosion of the Mg(OH)_2_ passivation layer can be effectively removed (Figure 9). This will expose a new Mg surface, facilitating the continuous and uninterrupted generation of hydrogen bubbles in the Mg-H_2_O reaction. This opens up possibilities for manufacturing diagnostic devices at the point of care on site, combined with autonomous mobile micromotors for rapid sample detection.

**Figure 10:**
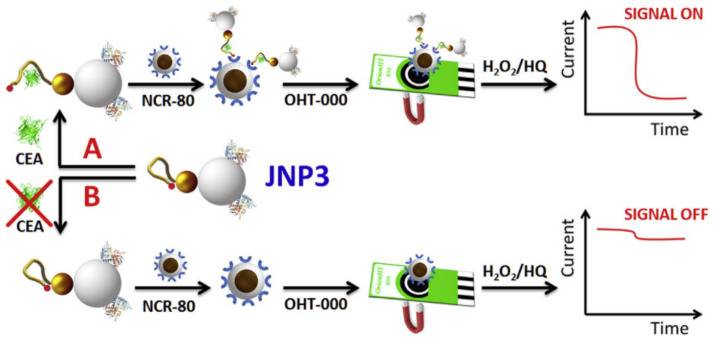
Schematic display of the Janus nanoparticles-based biosensing strategy for CEA detection. Reprinted with permission from ref. [157]. Copyright © 2019 Elsevier B.V.

Villalonga’s group measured carcinoembryonic antigen (CEA) with biofunctionalized JPs [157]. One of the most important biomarkers to monitor treatment for colorectal carcinoma and detect pancreatic, gastrointestinal, lung, ovarian, and breast malignancies is CEA. The silica surface of JPs was functionalized with HRP, and the Au surface was functionalized with a biotin thiol-specific hairpin structure aptamer (Figure 10). In the presence of CEA, the hairpin structural aptamer opens to release biotin residues, and the CEA–JNP complex can be trapped by affine-modified Fe_3_O_4_@SiO_2_ nanocaptures. In the presence of H_2_O_2_/hydroquinone (HQ), magnetic nanocomplexes were deposited on the working electrode surface of a screen-printed carbon electrode (SPCE) using magnetic attraction for amperometric detection, allowing the detection of 1.20 × 10^−12^ M CEA standards. This work can be used for the determination of serum samples. Recently, this group reported the preparation of a novel Janus nanoparticle based on asymmetrically functionalized gold colloids by using partially masked Fe_3_O_4_@SiO_2_ core-shell nanoparticles (nanocapsules) as masking tools and topologically selective surface modification of polyamide amine (PAMAM) dendrites as the primary capping material [158]. Then, horseradish peroxidase (HRP) was immobilized on the dendritic surface as a signal oxidoreductase. The resulting nanoparticles were used as a biometric/signaling element to construct an amperometric touchpiece with a sandwich-type structure for the specific detection of this cardiac biomarker, ranging from 10 pg·mL^−1^ to 1.0 ng·mL^−1^, with a detection limit of 3.1 pg·mL^−1^. The electroassay device also showed good specificity, reproducibility and stability and could be used to quantify CRP in recombinant human serum samples. Wang et al. designed a self-propelled Fe_3_O_4_@SiO_2_/Pt nanomotor to label antibody IgG for binding to immune proteins [159]. Core-shell Au@Ag nanotubes (Au@Ag NCs) were used as markers of mouse secondary antibody IgG to amplify the detection signal of IgG produced by Ag oxidation. The self-propelled motion of JPs accelerated the specific recognition of immune proteins in the solution between anti-IgG and IgG and formed the sandwich immune complex. Due to the presence of magnetic ferric tetroxide, immune complexes can be rapidly transferred and modified on electrodes for the rapid and simple preparation of immunosensors with the help of external magnetic fields. The immunosensor can detect IgG sensitively and quantitatively by means of double pulse voltammetry (DPV). Self-propelled motion based on nanomotion promotes the self-assembly of sandwich immune complexes and explores a new strategy for the rapid and sensitive detection of IgG.

Sentic et al. reported an asymmetric particle that uses electrochemiluminescence (ECL) to detect glucose [160]. Bipolar electrochemistry (BPE) offers the possibility of designing such asymmetrical particles straightforwardly, since simply applying an electric field throughout the solution can facilitate the escape of local oxygen or hydrogen, resulting in gas production to drive the movement of the particles. The oxidation of nicotinamide adenine dinucleotide produced by the luminescence group and the glucose oxidase decomposition substrate causes ECL’s luminous intensity to be positively correlated with glucose concentration. In the presence of glucose, ECL emission can be seen with the naked eye. A negative control experiment without glucose in the solution does not produce measurable light emission. The presence of glucose can be easily observed using this method, but its accurate content cannot be determined, and the detection limits are not exact.

## 4. Janus Particles for Optical POCT

Optical biosensors, becoming increasingly crucial in the sensing field [161], have been given a new design concept thanks to the advent of nanotechnology [162,163,164,165]. The combination of optical biosensors and nanotechnology [166,167] enables the traditional biosensor to detect the target more sensitively [168] and dramatically improves the intensity and durability of the detected signal [169]. These techniques must be used to achieve two different kinds of goals [170]. One involves identifying a small panel of specific protein biomarkers in numerous samples collected at various stages of diagnosis, prognosis, treatment, and monitoring [171,172,173]. The achievement of this objective is essential for personalized medicine, which provides appropriate care for each patient [174,175]. The other primary purpose is to identify specific biomarker panels in samples taken from a group of patients to aid in the early detection of diseases [176]. These studies aim to confirm existing biomarkers or identify new ones. Advances in the second category are anticipated to hasten POC’s early screening of high-risk patients [177]. This chapter focuses on applying JPs in fluorescence-based POCT and vision-based POCT.

### 4.1. Janus Particles for Fluorescence-Based POCT

Fluorescence biosensors are an optical biosensor type that is less expensive, faster to respond to requests, easier to use, and more sensitive and selective with regard to target molecules than classic biosensors [178,179,180]. The main advantage of fluorescent biosensors is that their construction requires only simple and inexpensive analytical equipment [181,182]. In situ screening and field detection can be carried out in the portable fluorescent biosensor [183]. When JPs are used for fluorescence detection, fluorescein-modified DNA or peptides and signal initiating devices can be modified on both sides of JPs, which can realize the separation, enrichment of biomarkers, signal transduction, and even realize the simultaneous detection of different analytes. Fluorescence biosensor constructed by JPs has the advantages of high sensitivity, strong anti-interference, rapid response, and simple detection procedures.

**Figure 11:**
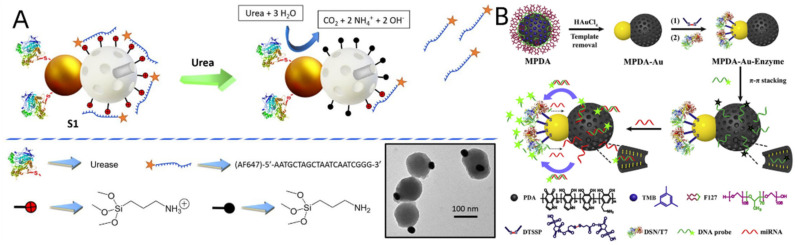
(**A**) Representation of the design and sensing performance of the nanosensor S1. Inset: TEM image of Janus Au--MSNPs. Reprinted with permission from ref. [184]. Copyright © 2019 Wiley--VCH. (**B**) Schematic illustration for the synthetic procedure and the miRNA detection application of Janus MPDA--Au nanoparticles. Reprinted with permission from ref. [185]. Copyright© 2020 Elsevier B.V.

Máez et al. introduced a brand-new bio-inspired nanoarchitectonics method to optical probe design [184]. In the Janus nanoparticles, the silica face was functionalized with amino moieties, which were positively charged at neutral pH and to which oligonucleotides adhere electrostatically. The fluorescent dye (Alexa Fluor 647) was labeled on the 5′ end of the oligonucleotide strand, containing 20 nucleotides. The urease enzyme was also immobilized using the gold face (Figure 11A). Enzyme-mediated hydrolyzed urea is converted to ammonia, which increases the pH of the resolution, and subsequent deprotonated amino groups on the silica surface enable the nanodevice to release fluorescent oligonucleotides. This straightforward nanodevice has been used to detect urea fluorimetrically in actual human blood samples and to identify tainted milk. Similarly, Tang et al. developed a biocomponent hybrid of JPs with immobilized enzymes for fluorescence-based “off-on” detection in solution [185]. Specifically, JPs consist of a mesopore polydopamine nanoparticle (MPDA) side and a gold (Au) particle side, where fluorescent DNA probes are modified on the MPDA side, and a duplex-specific nuclease (TSN)/T7 exonuclease (T7) is modified on the gold side to achieve substrate recognition and target cycling through the formation and hydrolysis of DNA hybrids (Figure 11B). The MPDA side acts as the “sensing unit” to direct the fluorescence recovery governed by target miRNAs binding and heteroduplex release, while the Au side is the “amplification unit” through the target recycling driven by the duplex cleavage via immobilized enzymes. Using miRNAs as model targets, the developed nanosensor achieves sensitive detection with a lower limit of 3.2 × 10^−14^ M (2.0 × 10^−14^–5.0 × 10^−13^ M).

**Figure 12:**
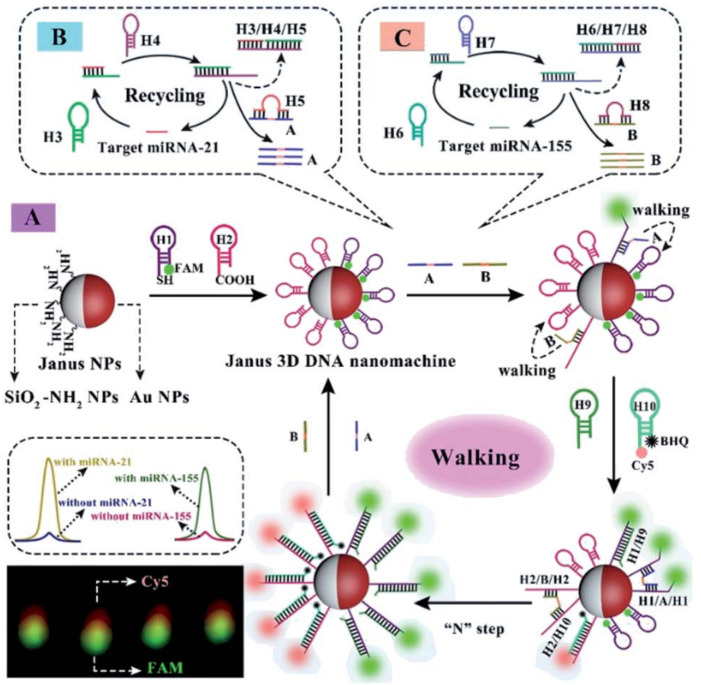
(**A**) Schematic illustration of the mechanical principle of the Janus 3D DNA nanomachine for simultaneous detection and sensitive fluorescence imaging of miRNA-21 and miRNA-(**B**,**C**) double feet catalyst strands A and B obtained by the conversion of target miRNA-21 and miRNA-155 with the help of catalytic hairpin assembly (CHA) DNA amplification, respectively. Reprinted with permission from ref. [186]. Copyright The Royal Society of Chemistry 2020.

Yang et al. constructed a Janus 3D DNA nanomachine to simultaneously detect and image dual miRNAs in cancer cells [186]. In this system, JPs were synthesized as a carrier to immobilize two different oligonucleotides on two different functionalized hemispheres to form Janus 3D DNA nanostructures, which transformed trace amounts of mirNA-21 and mirNA-155 targets into large amounts of FAM and CY5-labeled duplexes (Figure 12). Sensitive detection and imaging of miRNAs targets in cancer cells can be achieved by inducing two significant fluorescence emissions by catalytic hairpin assembly (CHA) and the 3D DNA cascade amplification strategy. Compared with the current miRNA imaging methods based on nanoparticle components, this method can effectively eliminate the “false positive” results, and the detection limit was down to 3.5 × 10^−13^ M and 4.8 × 10^−13^ M for miRNA-21 and miRNA-155 sensitive analysis. Meanwhile, the use of Janus nanoparticles as a carrier in the detection of single-type miRNAs to add two different fixed concentration signal probes further enhanced the fluorescence intensity. The proposed fluorescence imaging technology makes it possible to visualize low concentrations of miRNAs associated with minor changes in certain cancers, with significantly improved accuracy and precision compared to conventional fluorescence in situ hybridization methods.

**Figure 13:**
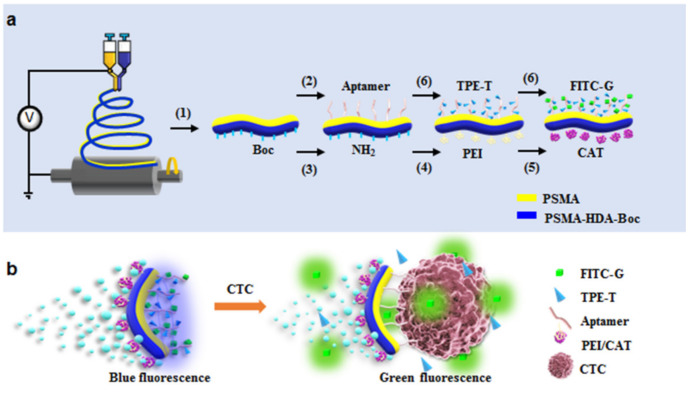
(**a**) Preparation of self-propelled JMs. (**b**) Ratiometric fluorescence response of JMs after the capture of CTCs. The binding between fluorophores with aptamers on JMs resulted in the ACQ effect of FITC-G and AIE of TPE-T, leading to blue fluorescence emission. In the presence of CTCs, the fluorescence of TPE-T was weakened rapidly, and that of FITC-G was restored to emit green fluorescence. Reprinted with permission from ref. [120]. Copyright © 2019 Elsevier B.V.

Identifying circulating tumor cells (CTCs) in peripheral blood helps track the development of the tumor, the effectiveness of available treatments, tumor recurrence, and patient survival. It should be noted that in 1 mL of blood, there are only a few CTCs, while there are a large number of hematological cells, such as about 5 × 10^9^ red blood cells, 1.0 × 10^7^ white blood cells, and 3.0 × 10^8^ platelets. Deficient levels in the blood pose a considerable challenge in developing ultra-sensitive CTC detection methods. To solve this problem, Xiaohong Li’s team combined a one-sided modified catalase Janus rod (JR), with the TLS11a aptamer coupled on the other side of JRs, and the tetrastyrene (TPE) derivative and fluorescein isothiocyanate (FITC) labeled on the aptamer by means of the interaction of base pairs [120]. JMs release TPE and FITC due to the competitive binding of tumor cells to aptamers, reducing the aggregation-induced emission (AIE) effect of TPEs and the aggregation-induced quenching (ACQ) spectrum of TPCs on JMs. As a result, after trapping tumor cells, Janus motors (JMs) exhibit a discernible shift in ratio fluorescence from blue (I450) to green (I526) (Figure 13). The fluorescence intensity ratio of I526/I450 can be matched to the cell level, with a detection limit of approximately 25 cells/mL in 1 min. Additionally, the recovery rate of HepG2 cells in blood samples was above 95%, indicating that this method can be utilized directly for the detection in the blood. The JM-2 detection of HepG2 did not interact with the presence of other tumor cells (4T1 and H22).

**Figure 14:**
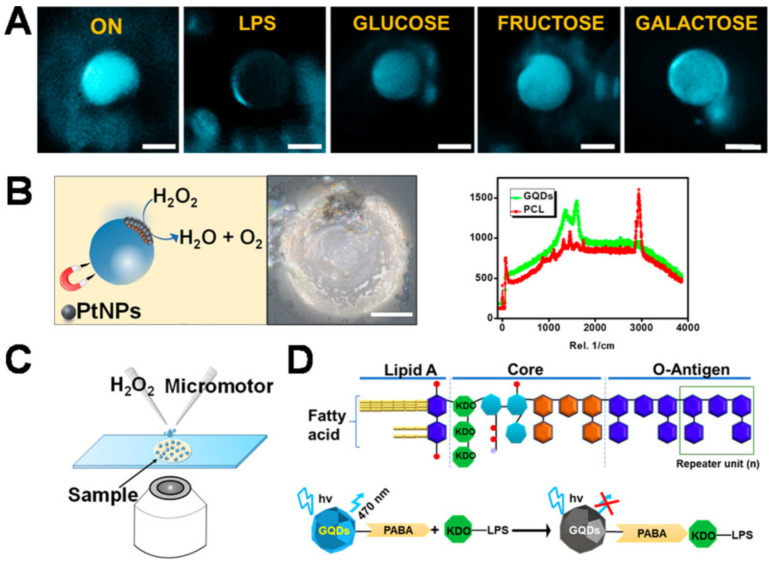
Self-propelled Janus microsensors for detecting LPS from Salmonella enterica (serotype enteritidis). (**A**) Real-time optical visualization of the LPS recognition event: time-lapse FL fluorescent images of the micromotors before (ON) and after LPS addition (OFF) and selectivity of the protocol in the presence of interfering saccharides. (**B**) Schematic of the microsensor operation and characterization. Optical images and Raman mapping show the distribution of nanoparticles, GQDs, and polycaprolactone (PCL) in the microsensor and corresponding Raman spectra. (**C**) Schematic of the setup for the Janus micromotor-based sensing protocol. (**D**) Schematics for the structure of the LPS from Salmonella enterica and mechanism of quenching by LPS union to the quantum dot (GQDs) recognition units contained in the microsensors. Reprinted with permission from ref. [126]. Copyright © 2018 American Chemical Society.

Lipopolysaccharide (LPS), a material made up of lipids and polysaccharides, is a part of the outer wall of the cell wall of Gram-negative bacteria (glycolipids) [187]. The research team of B. Jurado-Sanchez used Janus micromotors as portable sensors to identify toxins generated by Enterobacterium as signs of tainted food [126]. The Pickering emulsion process was used to create the micromotor, which depends on the simultaneous encapsulation of receptor functionalized quantum dots (QDs), which bind to specific sites in endotoxin molecules, and platinum nanoparticles to improve bubble propulsion. To interact with the quantum dots and cause the natural fluorescence of the micromotor to quickly quench in a concentration-dependent way, LPS was employed as the target endotoxin (Figure 14). Endotoxin concentrations as low as 0.07 ng/mL, far lower than the amount (275 g/mL) considered hazardous to humans, can be easily detected by micromotor testing. 

**Figure 15:**
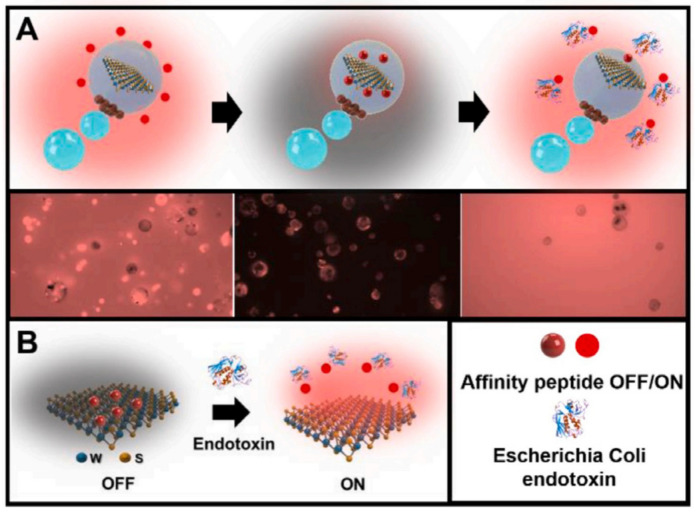
(**A**) Schematic of the Janus WS_2_-based micromotor strategy for *Escherichia coli* O111:B4 lipopolysaccharide (endotoxin) detection. From left to right: incubation with the specific affinity peptide (TMRholabeled); decrease in the fluorescence of the solution due to particular attachment to the WS_2_ material encapsulated in the micromotor and fluorescent recovery in the solution due to competitive interaction and specific release in the presence of the target endotoxin. The bottom part shows time-lapse fluorescent images of each step. (**B**) The sensing principle is based on the specific decrease in the fluorescence of the solution due to the attachment of the labeled peptide via electrostatic and hydrophobic interactions with the 2D material and subsequent recovery due to the generation of an endotoxin-fluorescent marked peptide complex and specific detachment in a concentration-dependent manner. Reprinted with permission from ref. [188]. Copyright ©2020 Elsevier BV.

Pacheco et al. utilized the affinity peptide of *E. coli* lipopolysaccharides with rhodamine to modify WS_2_ on asymmetric particles composed of Pt and Fe_2_O_3_ [188]. The presence of surface sulfide groups in WS_2_ made this “active sensing fraction” negatively charged and bound to positively charged affinity peptides through electrostatic and hydrophobic interactions, decreasing fluorescence intensity. The micromotor navigates in a 5 L sample for 5 min before the specific separation of the probe, which happens only in the presence of the target LPS, raising the fluorescence intensity of the solution (Figure 15). In lipopolysaccharides with a similar structure, no fluorescence recovery was seen (from Salmonella enterica). The LOD was 1.20 × 10^−10^ M, the lowest reported compared to similar strategies. Recently, the same group modified similar JPs with transition metal dihalides (TMDs) and rhodamine (RhO)-labeled timeless peptides for endotoxin detection in Salmonella enteric bacteria [189]. The OFF-ON strategy relies on the specific binding of the peptide to the TMD to induce fluorescence quenching (OFF state), which is restored due to the selective binding of endotoxin (ON state). The developed strategy was applied to the determination of Salmonella typhimurium endotoxin in serum with a highly sensitive molybdenum disulfide detection limit (LODs) of 2.0 µg/mL. No fluorescence recovery was observed in the presence of endotoxins with a similar structure, indicating the high selectivity and high bacterial specificity of the protocol.

**Figure 16:**
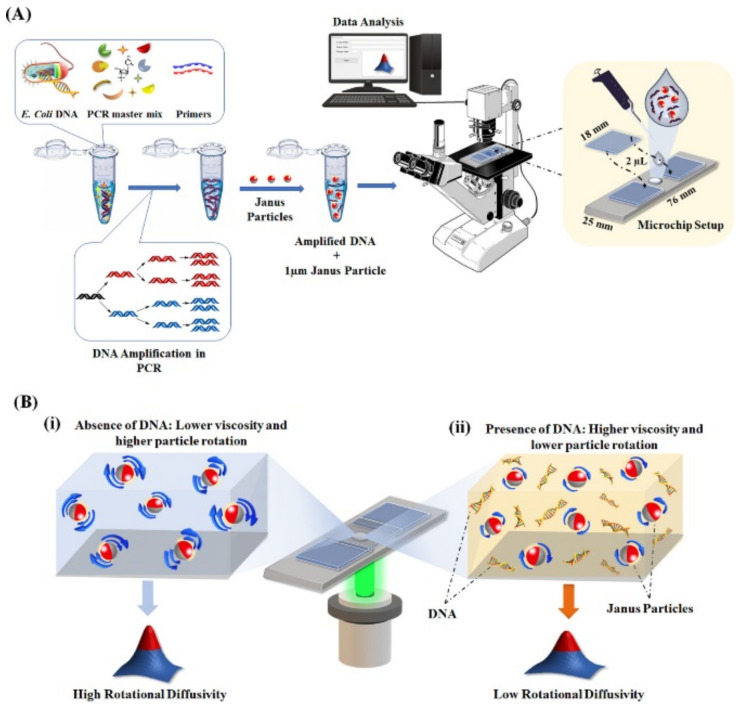
(**A**)Schematic illustration of the rotational diffusometry experimental setup. (**B**) Representative of rotational diffusometry (RD) in a microfluidic device with modified Janus particles. (i) In the absence of DNA, the Janus particles show higher rotational Brownian motion with a faster blinking signal. (ii) In the presence of DNA, the solution viscosity increases, which leads to a lower rotational Brownian motion of the particles and slower blinking signals. Reprinted with permission from ref. [191]. Copyright © 2021 American Chemical Society.

In addition to the above several detection methods, some unique rotational properties of JPs can also be used for POCT; for instance, Han-Sheng Chuang’s group proposed that according to the Stokes–Einstein–Debye relationship, the rotational Brownian motion has higher sensitivity than the translational change of the particle volume change. The rotational Brownian motion method detected TNF-α antigens by changing the particle diameter [190]. In the presence of TNF-α antigens, functionalized Janus particles form an intermediate immune complex to change the particle diameter. Tumor necrosis factor (TNF-α), interleukins, and interferons are usually released in the bloodstream to regulate exogenous invasions or endogenous abnormalities of the immune system; their presence may be an essential sign of certain infections or diseases. A cross-correlation algorithm determines the correlation intensity for two consecutive flickering images. Rotational diffusion is highly sensitive to the particle volume change and can be quantified by blinking signals. Compared to conventional ELISA, the rotation diffusion measurement method achieves a lower LOD (1 pg/mL) and higher resolution. Recently, the same research group used rotational diffusion (RD) to detect pathogens such as *E. coli* by expanding specific genes of Enterobacter [191]. When DNA was present in the solution, they utilized RD to monitor changes in the fluid’s viscosity. Compared to the same fluid without DNA, a rise in liquid density in the presence of DNA will produce reduced particle flicker and Brownian motion signals. Internal cross-correlation methods can be used to record and compute this reduction in flickering indications. The Janus particles they used were obtained by sputtering a thin layer of gold on fluorescently labeled polystyrene microspheres. A continuous flickering image of the modified beads was captured at 10 Hz with a CCD camera for 30 s (Figure 16). Then, cross-correlation algorithm analysis was applied to assess the degree of rotational diffusion. The drawbacks of sophisticated equipment and the lengthy real-time PCR analysis times (4–6 h) may be overcome using this technique.

Timothy M. Swager’s team described an emulsion-based agglutination technique for bacteria’s selective and sensitive identification [192]. The different hemispheres transformed into fluid Janus droplets, decisive liquid phase-detecting particles, when they were functionalized to exhibit orthogonal physical and biological features. At the surface of Janus droplets, mannose-containing surfactants self-assembled to form particles with lectin binding sites. Because the hydrocarbon and fluorocarbon solvents had different densities, Janus droplets were oriented vertically. Agglutination and a slanted geometry were caused by lectin binding to mannose(s). The apparent optical contrast between naturally aligned and agglutinated Janus droplets generates signals that could be quantified. The Janus emulsion assay was simple to prepare, has long-lasting stability, and binds to *E. coli* at 104 cfu/mL. A functional block copolymer surfactant that localizes at the boundary between the continuous phase of the droplets and their hydrocarbon phase was recently employed by the same research team [193]. This copolymer is composed of a hydrophilic polyacrylic acid block, a hydrophobic polystyrene block, and a polyacrylic acid block partly conjugated with trans cyclooctene (Poly-TCO). Listeria antibodies’ free amines were functionalized through a reaction with the NHS ester of tetrazine. In Janus droplets generated in PBS buffer with Poly-TCO localized at the hydrocarbon–continuous phase interface, tetrazine and trans cyclooctene experience an in situ bioorthogonal response. Agglutinations, collections of slanted droplets, are produced when the droplet-immobilized antibodies bind multivalently to Listeria.

**Figure 17:**
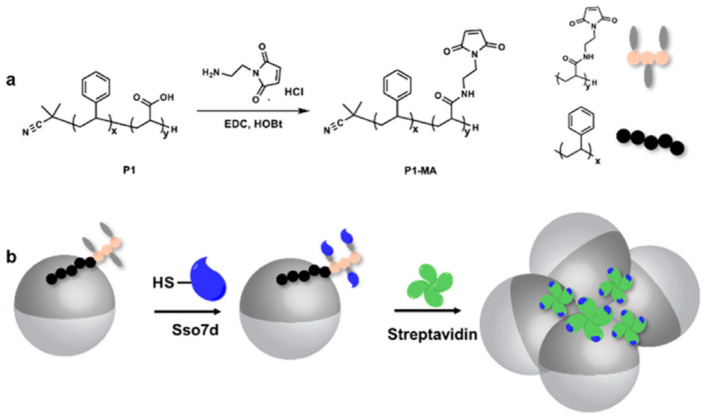
Functionalization of the droplets with the polymer surfactant. (**a**) Synthesis of maleimide functionalized surfactant P1-MA from a polystyrene-b-poly(acrylic acid) polymer. (**b**). Bioconjugation of rcSso7d to the droplet H/W interface via maleimide–thiol chemistry. The addition of streptavidin to the rcSso7d-functionalized droplets assay cause agglutination. The hydrocarbon phase is shown in dark gray for display purposes. Reprinted with permission from ref. [121] Copyright © 2019 American Chemical Society.

Additionally, the SARS-CoV-2 spike receptor-binding domain (RBD) [194] and IgG protein secondary antibody were used to functionalize the hydrocarbon/water interface, resulting in two distinct Janus emulsions [121]. A mixture of these Janus droplets could be detected in an agglutination test against SARS-CoV-2 spike IgG antibodies, which were caused by the binding of this antibody to the secondary antibody of the IgG antibody and the SARS-CoV-2 spike protein RBD. Within two hours, anti-SARS-CoV-2 spike antibody levels at concentrations as low as 0.2 g/mL could be found using qualitative optical pictures and quantitative fluorescence spectra. This was the basis for making inexpensive portable tools for prompt on-site infection detection. For the purpose of detecting the Zika NS1 protein, Janus emulsion droplets were functionalized with an antigen-binding protein rcSso7d variant that is thermally stable (rcSso7d-ZNS1) [195]. Fluorescent dye emulsion droplets increase the inherent optical signal in their fluorocarbon and hydrocarbon phases, which an essential optical fiber can detect (Figure 17). This assay offered a reliable, affordable, and practical method for the in-field detection of the Zika virus and other infections.

### 4.2. Janus Particles for Vision-Based POCT

Vision-based biosensors often do not require large instruments and equipment [196,197], and a small microscope [198,199] or a smartphone [200,201] can read out the detection signal through naked-eye observations [202,203,204]. Nano- or micromotors are inspired by natural movements, such as those of sperm, bacteria, or molecular motors, and are promising materials in many biomedical applications [205]. Compared to other types of motors, JPs have a chemically or physically unique anisotropic structure that can be easily modified [206]. In recent years, many reports have shown that Janus micromotors exhibit excellent performance or great potential in POCT. The asymmetric modification of JPs can provide a power source on one side when applied to vision-based bioassays, magnetic substances are usually introduced into the construction of JP to control the direction of motion, and Au, as a readily modified substrate, can modify aptamers to capture biomarkers. Therefore, a large number of magnetic JPs use Au as micro- and nanomotors to cause changes in motor speed or distance in the presence of targets, generating visual signals [122]. The asymmetry of JPs can power the movement of particles in complex samples, resulting in visual motion signals in a short period of time [207,208].

**Figure 18:**
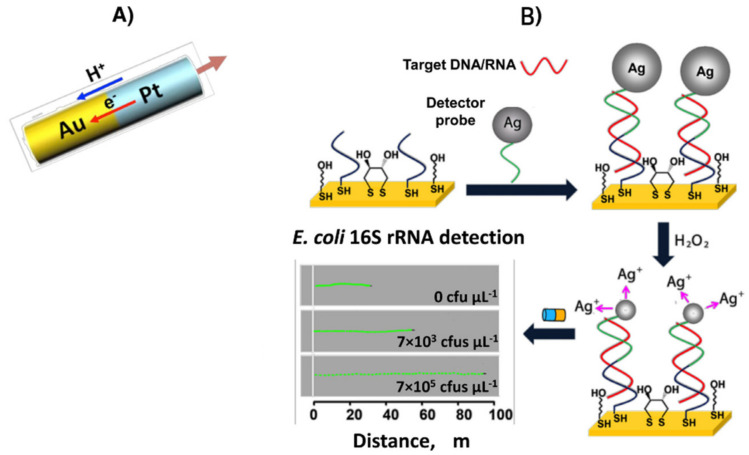
Pt-Au bisegment nanowires (**A**). Basis of the methodology developed for nucleic acid determination and visual detection of the movement of catalytic nanomotors in the resulting Ag^+^ enriched H_2_O_2_ solutions after hybridization with the complementary nucleic acid (**B**). Reprinted with permission from ref. [209]. Copyright © 2010, Nature Publishing Group.

Wang’s group invented the use of non-spherical Janus particles for optical (bio)sensing (Figure 18A). They demonstrated how to quickly, easily, and sensitively detect bacterial ribosomal RNA and DNA using synthetic nanomotors [209]. The motion-driven DNA-sensing concept uses a sandwich DNA hybridization test to capture tags made from silver nanoparticles that dissolve to measure changes in the speed of unmodified catalytic nanomotors (Figure 18B). With the aid of magnetically aligned “racing” nanomotors, optical microscopy was used to visualize the concentration-dependent distance signals. By adopting various motion transduction systems, this nanomotor detection strategy might be expanded to monitor different biomolecular interactions, making it a flexible and effective tool for identifying biological targets. This test utilized this emotionally driven analytical output to find bacteria and nucleic acids (*Escherichia coli*, *E. coli*). The target nucleic acid (synthetic DNA and *E. coli* 16S rRNA) was incubated with a surface-bound oligonucleotide probe in a standard sandwich hybridization test before being detected by a detector probe tagged with silver nanoparticles (Figure 18C). The hydrogen peroxide fuel and silver nanoparticles quickly dissolved, and the resulting combination was added to the nanomotors. LODs of 4.0 × 10^−17^ M (synthetic DNA) and 2000 colony forming units (cfus) L^−1^ of *E. coli* were made possible by silver-induced changes in the nanomotor speed, which resulted in a clearly defined dependence of the speed signal on the concentration of the nucleic acid target. By initially suggesting speed and distance as analytical readouts, these biosensing technologies signified a paradigm change in bioanalysis. Combining biomolecular interactions and motion transduction principles provided a new method for detecting a range of target biomolecules.

**Figure 19:**
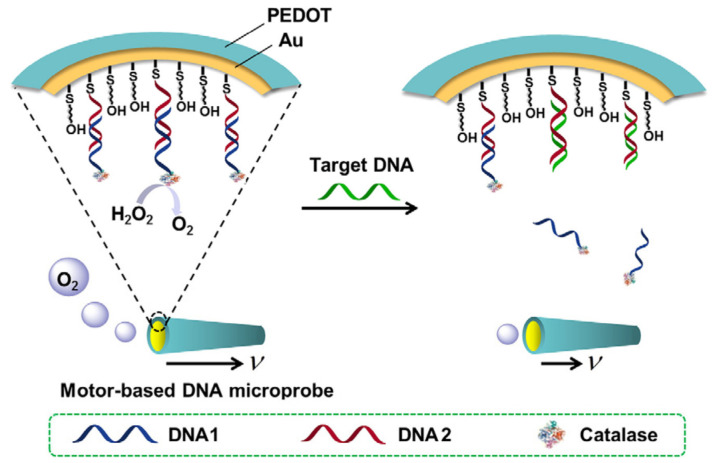
Schematic representation of motor-based microprobe for DNA detection via motion speed change. Reprinted with permission from ref. [210]. Copyright © 2016 Elsevier B.V.

Huangxian Ju’s team was the first to use catalase-modified Janus microtubules to detect DNA [123]. The immobilization of a specified sandwich DNA structure as a sensing unit on the PEDOT-PSS/Au, followed by the alternating hybridization with two aided DNA to bind the enzyme for effective motor motion, allowed for the assembly of the catalase. Under ideal circumstances, it showed speeds of up to 51 m/s in 0.25% H_2_O_2_ and 420 m/s in 2% H_2_O_2_. The sensing unit hybridized with the target DNA when it was present, releasing the multi-layer DNA and the multi-catalase and lowering the movement speed. The micromotor devised could detect DNA at concentrations between 1.0 × 10^−9^ M and 1 M by using speed as a signal. In subsequent research, a catalase layer was assembled on the inner surface of a poly(3,4-ethylene dioxythiophene)/Au (PEDOT/Au) microtube by the DNA conjugate, creating a motor-based microprobe [210]. The target molecule could control the microprobe’s motion signal, which propels the biocatalytic bubble (Figure 19). The target DNA displaced the DNA1-catalase conjugate, reducing the amount of assembled enzyme on the microtube and lowering the motion speed of the microprobe. Under ideal circumstances, the microprobe could quickly determine the concentration of a particular DNA in the range of 0.5–10 × 10^−3^ M without any washing or separating steps. This microprobe might be made in batches with good consistency and repeatability, and an optical microscope made it simple to see how quickly it moved. 

**Figure 20:**
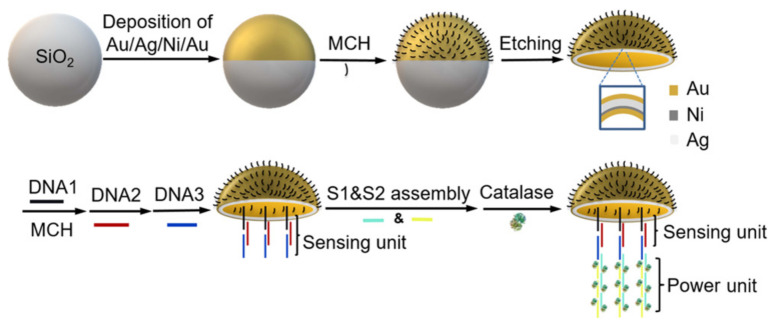
Schematic fabrication of the jellyfish-like micromotor. Reprinted with permission from ref. [124]. Copyright © 2019 American Chemical Society.

A multimetallic shell and DNA assembly with modified catalase decorations on the concave surface to mimic the umbrella-shaped body and the muscle fibers on the inner umbrella of a jellyfish were presented as the basis for a chemically propelled jellyfish-like micromotor [124]. The micromotor, which relies on catalase’s catalytic synthesis of oxygen gas in H_2_O_2_ fuel, demonstrated good bubble-propelled motion in various biomedia with speeds exceeding 209 m/s^−1^ in 1.5 % H_2_O_2_ (Figure 20). The jellyfish-like micromotors may be utilized to detect DNA motion based on the release of catalase via displacement hybridization. The “jellyfish-like” proposed micromotors demonstrated benefits, including precise manufacture, good motion capabilities, sensitive motion sensing of DNA, and strong stability and reproducibility, indicating significant promise for biological application. As a result, motion readout was used to create a straightforward and dynamic label-free detection approach for specific DNA fragments.

**Figure 21:**
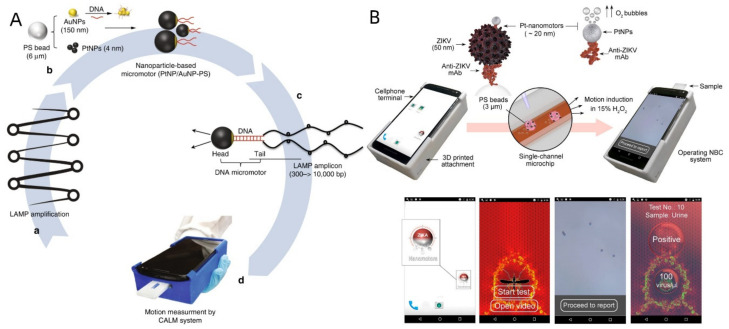
(**A**) Schematic presentation of HIV-1 detection using the cellphone system. The system integrates cellphone-based optical sensing, loop-mediated isothermal amplification, and micromotor motion (CALM). Reprinted with permission from ref. [211]. Copyright © 2018, the author(s). (**B**) Schematic of the NBC system for virus detection. Operating the NBC system loaded with a sample. Motion tracking application interface and data processing. Based on the detected change in the velocity of the beads, the concentration of the virus in the tested samples is detected, and a report for ZIKV infection is generated. Reprinted with permission from ref. [213]. Copyright © 2018 American Chemical Society.

A mobile phone-based HIV-1 molecular detection technique using loop-mediated isothermal amplification (LAMP) and micromotors (CALM) was presented by Draz et al. [211]. Significant health risks are posed by HIV-1 infection in industrialized and underdeveloped nations. The development of sensitive and portable technology for HIV-1 control may be made possible by combining mobile health strategies with bioengineered catalytic motors. The enormous stem-looped amplicons produced by LAMP amplification are predominantly used to modify the motion of DNA-engineered micromotors powered by metal NPs, such as platinum nanoparticles (PtNPs) and gold nanoparticles (AuNPs). When HIV-1 RNA was present in a sample, huge amplicons were formed, slowing down the motor speed (Figure 21A). With a cellphone system acting as the biomarker for target nucleic acid detection, the change in the motor’s motion might be precisely quantified. The offered technology enables sensitivity at a clinically meaningful threshold value of 1000 virus particles/mL. The cell phone network might allow viruses and other infectious diseases to be quickly and affordably diagnosed. 

A nanomotor-based bead motion cellphone (NBC) system for the immunological detection of ZIKV was later disclosed by this research team [212]. Platinum (Pt) nanomotors build up on the surface of the test sample’s beads due to viruses, which caused the beads to move in the presence of H_2_O_2_. The change in bead motion was then correlated with the viral concentration (Figure 21B). ZIKV could be found in samples with virus concentrations as low as one particle/liter using the recently established NBC method. The NBC method allowed for the precise detection of ZIKV in the presence of other neurotropic viruses, such as human cytomegalovirus, herpes simplex virus type 1, and the closely related dengue virus. Recently, Yuan et al. outlined a smartphone framework for the Janus micromotor that uses motion to detect glutathione [213]. A light-emitting diode (LED) source, a universal three-dimensional (3D) printed platform, and a commercial smartphone with an external magnification optical lens (20–400) that was directly attached to the camera made up the system. The sample holder could be adjusted to hold a glass slide. Due to thiol bond synthesis poisoning the catalytic layer, 20 nm graphene-wrapped/PtNPs Janus micromotors slow down when glutathione is present in peroxide-rich sample conditions. Speed and glutathione concentration can be connected, with a detection limit of 0.90 M, good selectivity, and percent recoveries even in interfering proteins and amino acids. The design of a test strip for quick glutathione detection (30 s) was made possible by naked-eye visualization of the speed decline, skipping earlier amplification techniques or sample preparation processes. This idea could be used for various micromotor strategies for future multiplexed methods that rely on fluorescence or colorimetric detection.

**Figure 22:**
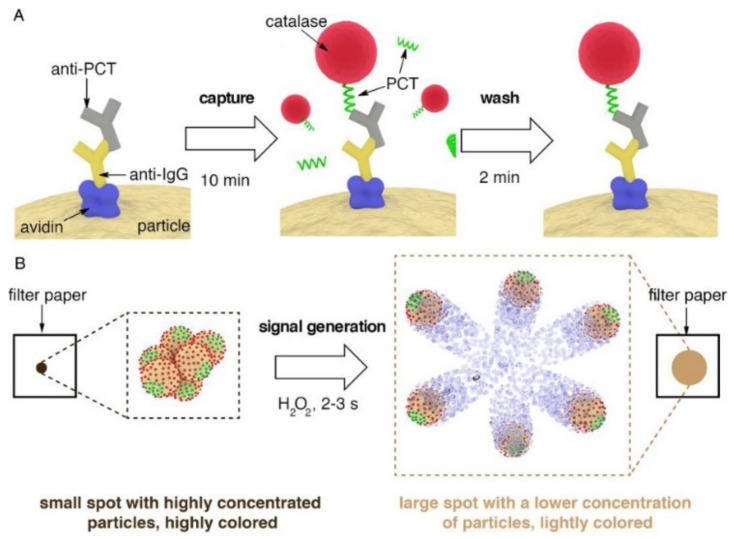
Schematic representation of the procedure for the rapid detection of PCT with the proposed multifunctional Janus particles; (**A**) PCT is specifically captured in blood utilizing a competitive immunoassay consisting of a 10 min capture step and a 2 min wash procedure; (**B**) signal generation mechanism: after spotting the particles on a piece of filter paper H_2_O_2_ is added; the catalase enzymes generate bubbles that propel the particles and disperse the color within seconds. The subsequent change in pixel intensity is read in real-time with a mobile phone app. Reprinted with permission from ref. [214]. Copyright © 2019 Elsevier B.V.

Using a novel motion-based signal generation technique, self-propelled particles revolutionize sensing applications by detecting biorecognition reactions as colloidal velocity changes. A new self-propelled, multipurpose Janus particle series that can see changes in particle velocity using colorimetry was described by Russell et al. [214]. These particles are made up of a Janus covering with biospecific identifying properties and an iron oxide core that gives them color and magnetic properties (Figure 22A). According to this technique, biomolecular interactions cause modifications in particle velocity that can be seen as color changes on a piece of paper. After that, these color variances are read and measured using a smartphone app (Figure 22B). Compared to other methods that require up to an hour-long incubation step under controlled conditions to find the same biomarker in purified serum, the proposed multifunctional particle biosensor design could detect the procalcitonin biomarker for sepsis in whole blood within 13 min at clinically significant concentrations.

**Figure 23:**
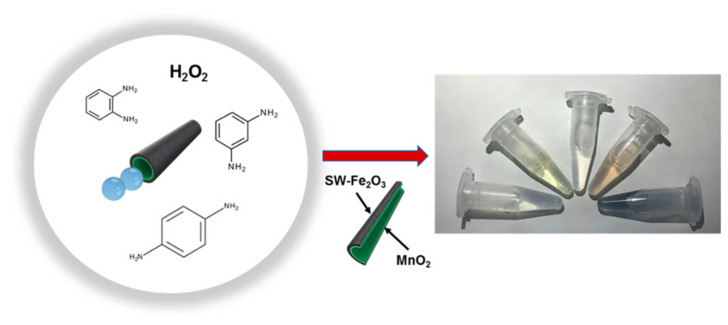
Schematic of the colorimetric assay with SW-Fe_2_O_3_/MnO_2_ micromotors for phenylenediamines detection. Reprinted with permission from ref. [79]. Copyright © 2018 American Chemical Society.

To detect and distinguish phenylenediamine isomers, B. Jurado-Sanchez’s team developed tubular micromotors comprising a driven catalyst of MnO_2_ and a hybrid single-wall carbon nanotube (SW) Fe_2_O_3_ outer layer [79]. Oxygen bubbles and hydroxyl radicals were produced during the catalytic decomposition of H_2_O_2_ as fuel, which was used in colorimetric assays to create colored solutions by dimerizing phenylenediamines (Figure 23). The Fe_2_O_3_ nanoparticles and the uneven SW backbone produced a rough catalytic layer with an increased hydroxyl radical generation rate and analytical sensitivity. These self-propelled micromotors function as mobile platforms resembling peroxidases, providing effective phenylenediamines detection and discrimination in just 15 min. For o-phenylenediamine and phenylenediamine, low detection limits (5 × 10^−3^ and 6 × 10^−3^ M, respectively) were obtained.

**Figure 24:**
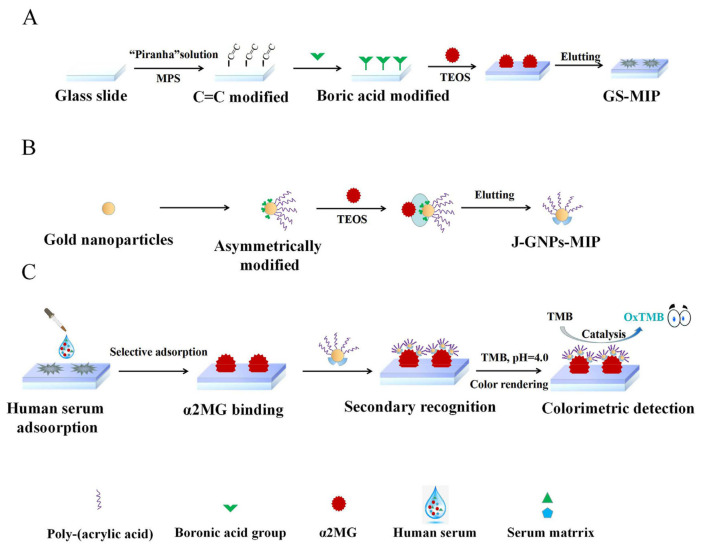
A synthesis scheme of the GS-MIP (**A**) and J-GNPs-MIP (**B**) and the detection mechanism of DMI-ICS (**C**). Reprinted with permission from ref. [215]. Copyright © 2022 Elsevier B.V.

Zhang et al. reported a dual molecular imprinting immunosandwich colorimeric approach (DMI-ICS) constructed to detect alpha-2-macroglobulin (a2MG) using Janus imprinted nanoparticles [215]. A2MG glass slides molecularly imprinted material (GS-MIP) was used in the first section as a “separation antibody” that can quickly and accurately separate the protein in a complicated sample. The other component was an asymmetrically modified Janus molecularly imprinted gold nanoparticles enzyme (J-GNPsMIP) that functions as a “detection antibody” and has the qualities of selective identification and catalytic substrate color (Figure 24). One may determine the concentration of a2MG by watching the fluctuations in substrate color. The DMI-ICS performed exceptionally well and exhibited a minor relative standard deviation, good linear range (0.297–130 mg/mL), high imprinting factor, and lower detection limit (0.089 mg/mL) in the optimal conditions.

Zeng et al. created an ultrasensitive colorimetric approach for the detection of biothiols based on the design of the Janus Pd-Fe_3_O_4_ enzyme with the use of density functional theory [216]. The Pd-Fe_3_O_4_ dumbbell-like nanoparticles (DBNPs), which were produced using seeds, showed a consistent heterodimeric nanostructure. Ultrasensitive biothiols were found using two different techniques. On the one hand, Pd-Fe_3_O_4_ DBNPs showed stronger peroxidase-mimicking activity than the individual components because of the synergistic effects of Pd and Fe_3_O_4_ in the dumbbell structure. On the other hand, the presence of the target biothiols molecules dramatically enhanced their ability to block the Janus Pd-Fe_3_O_4_ nanozyme. Both experiments and theoretical computing were used to validate the results mentioned above. A simple, highly selective, ultrasensitive colorimetric, and quantitative test for biothiols was developed based on a rational design. A maximum detectable amount (LOD) of less than 3.1 × 10^−9^ M was observed. The biothiols in actual urine samples were successfully identified using this method as well. Additionally, the Pd-Fe_3_O_4_ nanozyme was used to differentiate between biothiol levels in healthy and susceptible cancer cells at a cell density of 1.5 × 10^4^/mL. Demonstrating its significant potential in biological and clinical investigation. In addition to presenting the exceptional capabilities of Janus bimetallic nanozymes, this work offers logical directions for developing high-performance enzymes for use in biomedical and sensing systems.

So far, scholars have developed a variety of biomarker detection methods and JPs for POCT. Table 2 lists the different sensing schemes for POCT. It can be seen that the current development trend of JPs in POCT is to reduce the detection limit and improve the sensitivity through various strategies, break the limitations of large equipment and professionals, and achieve a high degree of sensitivity and portability of POCT.

**Table 2:** Different sensing schemes for POCT.

Detection Method	Analyte	Compositions	Type	Sensitivity	LoD	Ref.
Electrochemical biorecognition-signaling	Glucose	Au-SiO_2_	Enzymesensor	490 nM–600 mM	360 nm	[154]
		Mg/Pt Janus micromotors	Non-enzymesensor	1–15 mM	33.2 μM	[154]
	Ochratoxin A	Au@SiO_2_	Non-enzymesensor	1 × 10^−5^–10 nM	3.3 × 10^−3^ pM	[155]
	Carcinoembryonic antigen	Fe_3_O_4_@SiO_2_	Non-enzymesensor	5.5 pm to 28 nM	1.2 pM	[157]
	C-reactive protein	Fe_3_O_4_@SiO_2_-Au	Enzymesensor	10 pg/mL–1.0 ng/mL	3.1 pg/mL	[158]
	IgG	Fe_3_O_4_@SiO_2_/Pt	Non-enzymesensor	10 pg/mL to 100 ng/mL	3.14 pg/mL	[159]
Electrogenerated chemiluminescence	Glucose	glassy carbon	Enzymesensor			[160]
Flurorimetric detection	Urea	Au- SiO_2_	Enzymesensor	1.25–8.75 mM	0.5 mM	[184]
	MicroRNA	Au-MPDA	Enzymesensor	20–500 fM	32 fM	[185]
		Au@SiO_2_	Non-enzymesensor	1 pM–10 nM	0.35 pM	[186]
	circulating tumor cells	Janus fibers	Non-enzymesensor	0–10^6^ cells/mL	25 cells/mL	[120]
	Lipopolysaccharide	Graphene quantum dots	Non-enzymesensor	0.2–1.0 ng/mL	0.07 ng/mL	[126]
		WS_2_–Pt–Fe_2_O_3_ polycaprolactone	Non-enzymesensor	4.0–1.0 × 10^6^ ng/mL	120 pM	[188]
	Salmonella enterica endotoxin	WS_2_–Pt–Fe_2_O_3_	Non-enzymesensor	4–333.3 µg/mL	2.0 µg/mL	[189]
		MoS_2_–Pt–Fe_2_O_3_	Non-enzymesensor	9.8–333.3 µg/mL	2.0 µg/mL	[189]
Emulsion agglutination assay	Tumor necrosis factors alpha	Au@SiO_2_	Non-enzymesensor	1 pg/mL–10 μg/mL	1 pg/mL	[190]
	DNA	Au@SiO_2_	Non-enzymesensor		0.1ng/μL	[191]
	The anti-SARS-CoV-2 spike IgG antibody	Hydrocarbon and fluorocarbon oils	Non-enzymesensor		0.2 μg/mL	[121]
	Zika NS1 protein	Hydrocarbon and fluorocarbon phases	Non-enzymesensor		100 nM	[195]
Motion-based detection	DNA	Au–Ni–Au–Pt nanomotors	Enzymesensor	100 pM–10 nM	10 pM	[209]
		PEDOT-PSS/Au	Enzymesensor	10 nM–1 μM		[123]
		PEDOT/Au	Enzymesensor	0.5–10 mM		[210]
		Au/Ag/Ni/Au	Enzymesensor	25–750 nM		[124]
	HIV-1 RNA	Pt/Au@PS	Non-enzymesensor			[211]
	Zika virus	Pt@PS	Non-enzymesensor	100 particles/μL–10^6^ particles/μL	1 particle/μL	[212]
	Glutathione	Graphene-wrapped/PtNPs	Non-enzymesensor	5–150 μM	0.90 μM	[213]
Colorimetric detection	Aspartic Acid	Janus AuNPs	Non-enzymesensor	18 μM–1.8 nM	33.9 μM	[55]
	Procalcitonin	Magnetic beads	Non-enzymesensor		2 ng/mL	[214]
	Phenylenediamines Isomers	single-wall carbon nanotube (SW)-Fe_2_O_3_	Non-enzymesensor		20 μM	[79]
	Alpha-2-macroglobulin	Janus AuNPs	Non-enzymesensor	0.297–130 mg/mL	0.089 mg/mL	[215]

## 5. General Conclusions, Challenges, and Perspectives

Here, we reviewed the special characteristics of JPs as well as currently prevalent design and construction techniques. We also focused on the potential application of JPs in POCT and assessed the advantages of JPs as new biosensors. JPs have different physicochemical properties, morphological asymmetry, and spatially discrete surface domains. JPs, a unique class of materials, are now of great research interest for the synthesis of tailored materials and a variety of biological applications. JPs have been manufactured using a variety of synthesis techniques up to this point, including the microfluidic method, the sputtering method, the phase-separation method, and the Pickering emulsion method. In addition, the unique surface chemistry of JPs provides a simple modification method for coupling multifunctional moieties and has been widely used to target vectors and multifunctional cellular delivery. Even in the most recent synthesis techniques, it is still challenging to accurately manage the morphology of JPs during mass production. To achieve a reasonable design and synthesis of Janus nanoparticles, further studies are needed to investigate the assembly mechanism between different materials. It is clear that to make the big jump from the lab to the market, researchers must standardize and improve manufacturing for more consistent outcomes. They must next look towards incorporating more power sources to improve their long-range navigation and deep penetration, enabling fully autonomous operation under challenging circumstances.

In terms of applications, the asymmetry of JPs and the corresponding anisotropic structure can reduce the detection time of JPs in POCT and overcome the influence of a viscous environment. For example, JPs can be modified to capture biomarkers on one side and to provide propulsion on the other side so that testing can be conducted quickly, even in complex body fluid samples. Although JPs have shown promising advantages in biological detection, more application studies are needed to demonstrate the improved properties of JPs compared to those of traditional homogeneous nanoparticles in POCT. The study of JPs in POCT is still in its early stages, although promising results have been achieved. During electrochemical detection, JPs modified with capture units are deposited on electrodes to generate electrochemical signals in the presence of targets, but this process usually relies on electrochemical workstations and requires complex elution processes. In the process of fluorescence-based detection, a fluorescence microscope and other pieces of equipment are needed to further process the fluorescence signal. The lack of convenient characterization technology is a challenge in the application of JPs in POCT. To improve this situation, research in recent years has focused on smartphone-based and visual biometric detection. Visual detection based on the motion of JPs is the most accessible method, but the movement of the JPs is without direction, leading to the inconvenience of the detection process. In addition, although Janus nanoparticles with different compositions and morphologies have been reported in previous studies, the availability of JPs for biomarker detection of novel probes is very limited. As a result, more JPs with advanced environmental friendliness, enhanced controllability, signal processing, and conversion performance are expected. The synthesis of multimodal targeting/imaging JPs with different optical, magnetic, or rotational dynamics is still the trend of the times. Finally, additional studies are needed to evaluate the environmental friendliness and portability of each type of JPs for successful clinical translation. It can be predicted that in the next few years, studies in JPs will show a significant increase in portable electrical analysis systems integrated into smartphones or wearable sensors for rapid biosensing.

Briefly, based on the design, preparation, and POCT applications, morphology, composition, and surface modification will affect the application of JPs, some of which have shown very good performance in biosensing compared to conventional homogeneous particles. There is still a long way to go in developing simple, large-scale manufacturing strategies, building biodegradable and low-toxicity JPs, designing JPs with self-contained and self-controlling properties, and portable JP-based detection devices. Here, we hope that this review will provide a better understanding of the design and preparation of JPs, while further stimulating interest in expanding POCT applications.

## Figures and Tables

**Table 1:** Synthesis method, morphology and particle size of Janus nanoparticles.

Synthetic Methods	Compositions	Morphology	Particle Size (µm)	Application	Ref.
Microfluidic method	Polyurethane	Spherical	40–100		[62]
	Polymer poly(lactic-co-glycolic acid)	Spherical	0.3	Drug delivery	[80]
	PSMA/PS	Fibriform	1.9–20	Biological detection	[120]
	Hydrocarbon and fluorocarbon oils	Droplet-shaped	20	Biological detection	[121]
Sputtering method	Au@SiO_2_	Spherical	0.4		[87]
	Pt@SiO_2_	Spherical	0.5	Drug delivery	[122]
	SiO_2_	Spherical	0.1	Drug delivery	[116]
	PEDOT-PSS/Au	Tubular	13.5	Biological detection	[123]
	Au/Ag/Ni/Au	Jellyfish-shaped	20	Biological detection	[124]
	Au/PEDOT/Pt	Tubular	12	Medical imaging	[11]
Phase-separation method	PVP-Fe_3_O_4_	Irregular spherical	8.7 × 10^3^		[98]
	Polystyrene(PS)/PMMA	Capped spherical	10		[99]
	Au@SiO_2_	Spherical	0.45		[100]
	Au-SiO_2_	Snowman-shaped	0.1	Biological detection	[125]
	PEG-CuS-Au-MnO_2_	Snowman-shaped	0.125	Imaging and therapy	[44]
	AuNPs	Spherical	1.3–4.5 × 10^−2^	Biological detection	[55]
	Fe3O4@PS/PGMA	Spherical	18–30		[90]
	Au/Fe_3_O_4_@C	Snowman-shaped	0.12	Dual-modal imaging	[46]
Pickering emulsion method	Molten paraffin	Spherical	1.5		[108]
	Toluene-SiO_2_	Spherical	0.45		[109]
	Graphene quantum dots	Spherical	20	Biological detection	[126]

## Data Availability

Not applicable.
